# Management of Acute Coronary Syndrome With a Bleeding Intra-Tumoral Hepatocellular Carcinoma: A Case Report

**DOI:** 10.7759/cureus.27653

**Published:** 2022-08-03

**Authors:** Ganesh Manudhane, Ria Mehta, Shamshersingh Chauhan, Maharudra Kumbhar

**Affiliations:** 1 Cardiology, SevenHills Hospital, Mumbai, IND; 2 Internal Medicine, SevenHills Hospital, Mumbai, IND; 3 Gastroenterology and Hepatology, SevenHills Hospital, Mumbai, IND

**Keywords:** therapeutic embolization, percutaneous transluminal angioplasty, pulmonary edema, incidental discovery, hepatocellular cancer, coronary angiography, bleeding

## Abstract

This case report presents the management of a 69-year-old gentleman with acute coronary syndrome in the setting of an incidentally detected hepatocellular carcinoma with intra-tumoral bleed. Initially, the patient presented with fever, cough, and sudden onset of dyspnea on rest accompanied by angina, after which he was diagnosed with non-ST segment elevated myocardial infarction complicated with congestive cardiac failure. His laboratory and radiological investigations were suggestive of a possible infective etiology which, in an era of COVID-19, was investigated further with a high-resolution CT scan of the chest, which was suggestive of features of pulmonary edema along with an incidental discovery of liver lesions on the abdominal cuts. A further workup with a dedicated triple-phase computed tomography scan abdomen demonstrated features of undiagnosed hepatocellular cancer with intra-tumoral bleeding. Therefore, a mesenteric celiac angiogram followed by trans arterial bland embolization of the bleeding vessel was performed. In the same setting, for the simultaneous management of the acute coronary syndrome, coronary angiography performed revealed a triple vessel disease which was immediately followed by a percutaneous transluminal coronary angioplasty.

## Introduction

There is a rising trend in the incidence of liver cancer, with an expected incidence of more than one million cases by the year 2025, out of which hepatocellular carcinoma (HCC) accounts for approximately 90% of cases [1]. The incidence is highly variable according to age, sex, comorbidities, and geographic location. Higher incidences are observed in males, with a peak around 70 years of age, especially in Asians and Black Sub-Saharan Africans [2]. Most of the cases are symptomatic, with occasional incidental cases discovered during imaging [3]. Spontaneous bleeding from hepatocellular carcinoma is a common complication and is among the common causes of death due to HCC [4]. Hepatic artery embolization is the line of management of such conditions and antiplatelets and anticoagulation are usually contraindicated. In this case, we come across a unique scenario of the management of an incidentally detected hepatocellular carcinoma with an active intra-tumoral bleeding, along with simultaneously administering anticoagulation and dual antiplatelets (DAPTs) for an invasive coronary angiography (CAG) followed by percutaneous transluminal coronary angioplasty (PTCA) to manage acute coronary syndrome.

## Case presentation

We report the case of a 69-year-old gentleman who presented to the emergency department of our hospital, with fever, cough, and sudden-onset breathlessness on rest, accompanied by angina, after getting transferred from another hospital on non-invasive ventilation. The patient initially presented to an external hospital where he was vitally unstable with a temperature of 100.2F, Saturation of peripheral oxygen (SpO2) measuring 84% on room air, and blood pressure (BP) of 90/50mmHg, after which he was vitally stabilized and transferred to our hospital on non-invasive ventilation. On admission, he had a heart rate of 130bpm, BP of 100/70mmhg, and SpO2 measuring 93% on room air. An electrocardiogram (ECG) coupled with a high sensitivity troponin T(hs-TnT) value of 217ng/L (normal value-<15ng/L ) done at the hospital, was suggestive of a non-ST segment elevated myocardial infarction (NSTEMI), complicated with clinical features suggestive of congestive cardiac failure (CCF) [5]. The patient denied any complaints of night sweats, weight loss, nausea, vomiting, or any change in bowel bladder habits. The patient was a known case of diabetes mellitus type 2, hypertension, chronic obstructive pulmonary disease, and peripheral vascular disease, and had a surgical history of left below-knee amputation and right lower limb angioplasty. His medications at the time of admission are summarized in Table 1.

**Table 1:** Medications taken at the time of hospitalization

Medication	Dosage	Frequency
Tab Ecosprin	75mg	Once a day
Tab Rosuvastatin	20mg	Once a day
Tab Doxophyllin	400mg	Twice a day
Tab Glimepiride	1mg	Once a day
Tab Vildagliptin +metformin	50mg/500mg	Twice a day
Tab Amlodipine	5mg	Once a day

**Figure 1:**
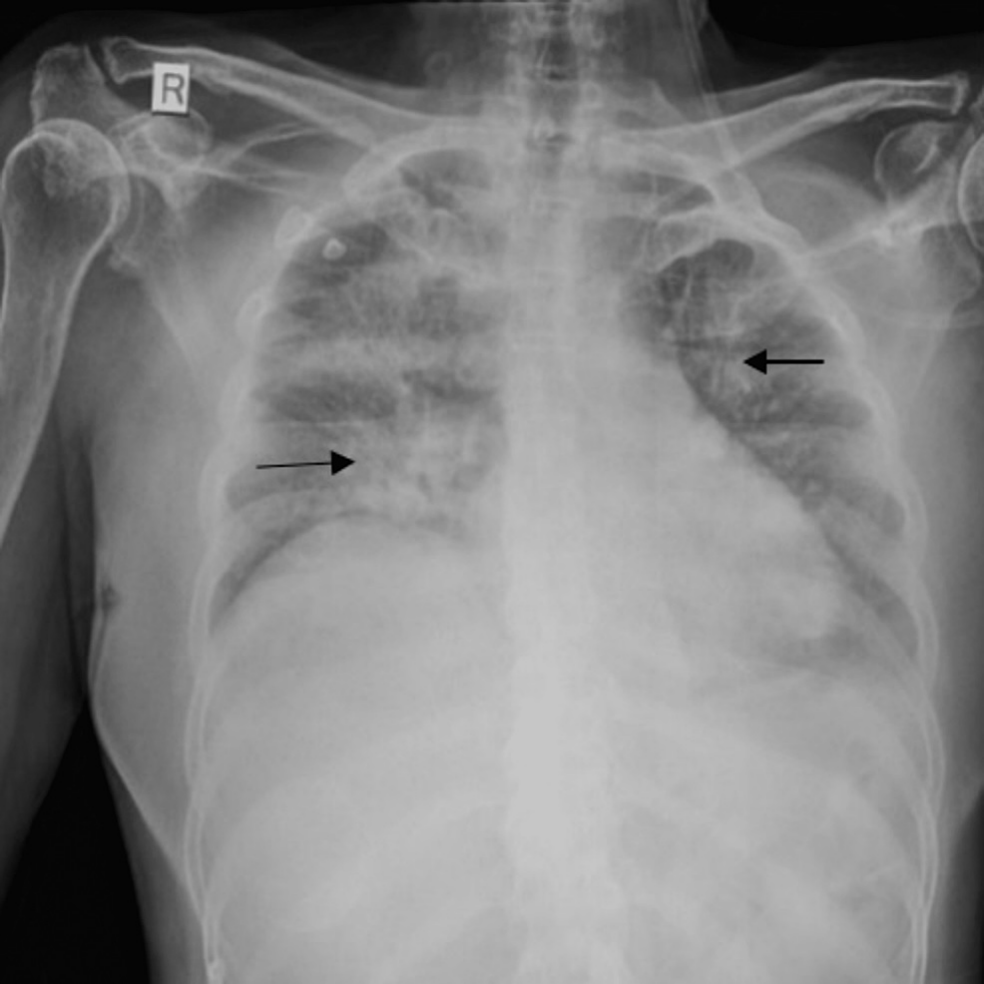
X-ray of the chest showing diffuse bronchopneumonia in right and left lungs (see arrows)

Given his symptoms in an era of the COVID-19 pandemic, a nasopharyngeal swab was collected and tested for COVID-19, whose results arrived a day after admission and were negative. His blood sample was collected and sent for routine laboratory investigations. The results of laboratory results are summarized in Tables 2-6. On admission, due to the presence of a fever spike along with productive cough, and raised blood counts, a chest X-ray was performed which was suggestive of findings of diffuse bronchopneumonia (Figure 1).

**Table 2:** Complete blood count WBC: white blood cell; RBC: red blood cell; PCV: packed cell volume; MCH: mean corpuscular hemoglobin; MCHC: mean corpuscular hemoglobin concentration

TEST NAME	RESULT	UNIT	REFERENCE
Total WBC count	15.59	X 10^3/ul	4.00-10.00
Neutrophils	74.9	%	40.00-80.00
Lymphocyte	14.4	%	20.00-40.00
Eosinophils	0.6	%	1.00-6.00
Monocytes	10.1	%	2.00-10.00
Basophils	0.0	%	1.00-2.00
RBCs	3.79	X 10^6/ul	4.50-5.50
Hemoglobin	10.2	Gm/dl	11.00-17.00
PCV	36.2	%	40.00-50.00
MCV	95.4	Fl	83.00-101.00
MCH	33.5	pg	27.00-32.00
MCHC	35.1	gm/dl	31.50-34.50
Red Cell Distribution Width-CV (RDW-CV)	14.1	%	35.00-46.00
Platelet	237	X10^3/ul	150.00-450.00

**Table 3:** Renal function tests BUN: blood urea nitrogen

TEST NAME	RESULT	UNIT	REFERENCE
Urea	46.22	mg/dl	10-50
BUN	21.60	mg/dl	6-20
Creatinine	0.79	mg/dl	0.5-1.5

**Table 4:** Electrolytes: serum

TEST NAME	RESULT	UNIT	REFERENCE RANGE
Sodium	135	mEq/L	135-148
Potassium	3.6	mEq/L	3.5-5.5
Chloride	98	mEq/L	96-106

**Table 5:** Liver function tests SGOT: Serum glutamic oxaloacetic transaminase; SGPT: serum glutamic pyruvic transaminase; GGT: gamma glutamyl transferase

TEST NAME	RESULT	UNIT	REFERENCE RANGE
SGOT (aspartate transaminase)	57.9	U/L	0-40
SGPT (alanine transaminase)	56.2	U/L	0-40
Total bilirubin	1.28	mg/dl	0-1.1
Direct bilirubin	0.63	mg/dl	0-0.6
Indirect bilirubin	0.65	mg/dl	0.1-0.8
Alkaline phosphatase	92	U/L	42-128
Total protein	5.85	gm/dl	6.3-8.4
Albumin	3.46	gm/dl	3.5-5.2
Globulin	2.39	gm/dl	2-4
GGT	77	U/L	10-71

**Table 6:** Coagulation profile

TEST NAME	RESULT	REFERENCE RANGE
Prothrombin time	13.1	Seconds
Control value	13.3	Seconds
Prothrombin index	101.53	%
Prothrombin ratio	0.98	Ratio
International normalized ratio (INR)	0.98	Ratio

**Figure 2:**
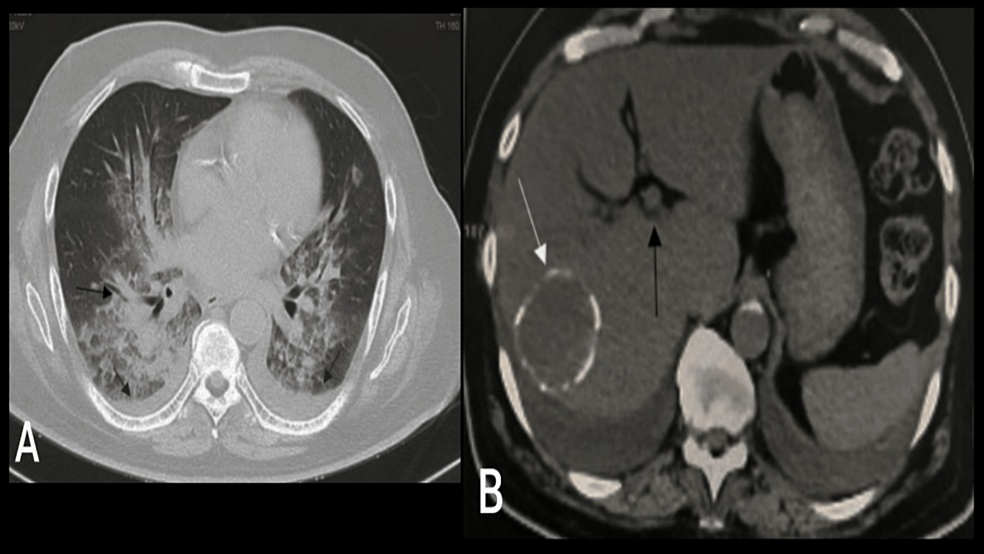
High resolution computed tomography of the chest taken on Day 2 of hospitalization (A) Bilateral perihilar consolidations intermixed with ground glass opacities (black arrows) with bilateral pleural effusion (blue arrows) and pericardial effusion. (B) Abdominal cuts showing unidentified liver lesions (white and black arrows).

In light of the COVID-19 pandemic, we performed a further workup with a high-resolution computed tomography (HRCT) of the chest which was suggestive of features of fluid overload (Figure 2A), along with an incidental discovery of liver lesions on the abdominal cuts (Figure 2B).

**Figure 3:**
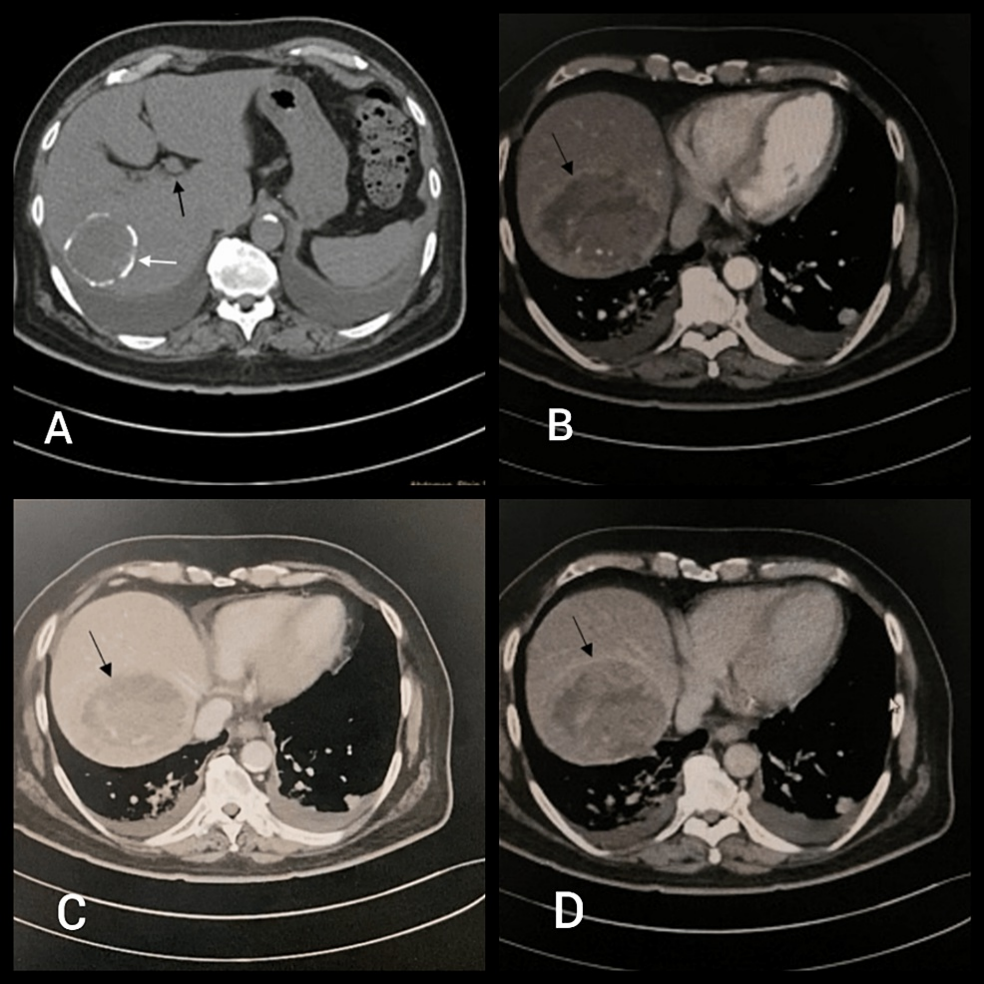
Triple-phase CECT of the abdomen (A) two hypodense masses in segments VII and VIII of the liver (black arrow) and a hydatid cyst in segment VII (white arrow); (B) late arterial phase with (C) washout on venous (D) delayed phase scans showing the appearance of peripheral capsular enhancement on delayed phase scans

A further workup with a dedicated triple-phase contrast-enhanced computed tomography (CECT) scan of the abdomen was hence performed, which showed a well-defined heterogeneously hypodense round mass, measuring 7.3x8.8x7.1cm, closely approximated to the diaphragmatic surface of the liver, with hyperdense areas within, which represented blood contents in segment VIII of the liver along with an incidental finding of a hydatid cyst measuring 4.5x4.8x4.2cm in the right lobe of liver segment VIII (Figure 3A). The lesion was enhanced in the late arterial phase with washout on venous, delayed phase scans such that there was an appearance of peripheral capsular enhancement on delayed phase scans (Figures 3B-D). The blood vessels coursing through the lesion on late arterial phase images were representative of branches of the hepatic artery. Another heterogeneous hypodense lesion measuring 3.1X3.3.x3cm was also noted in segment VII of the liver. The above findings, along with, enlarged necrotic periportal lymph nodes, were suggestive of a probable hepatocellular carcinoma [6] with regional nodal metastasis [7]. There was no evidence of liver nodularity on imaging. Any history of alcohol abuse, drug use, or any known chronic liver disease was denied by the patient. He tested negative for human immunodeficiency virus (HIV), hepatitis B surface antigen (HBsAg), and antibodies to hepatitis B core antigen (anti-HBc). His alfa-fetoprotein level was 1012 ng/ml (normal range: 0-40ng/ml). His Eastern Cooperative Oncology Group (ECOG) status prior to developing NSTEMI was 0. According to the staging system, his Child-Turcotte-Pugh (CTP) score was A [8], the model for end-stage liver disease (MELD) was 13 [9] and, the Barcelona clinic liver cancer (BCLC) staging was C [10], in view of the regional nodal metastasis at the time of presentation. 

**Figure 4:**
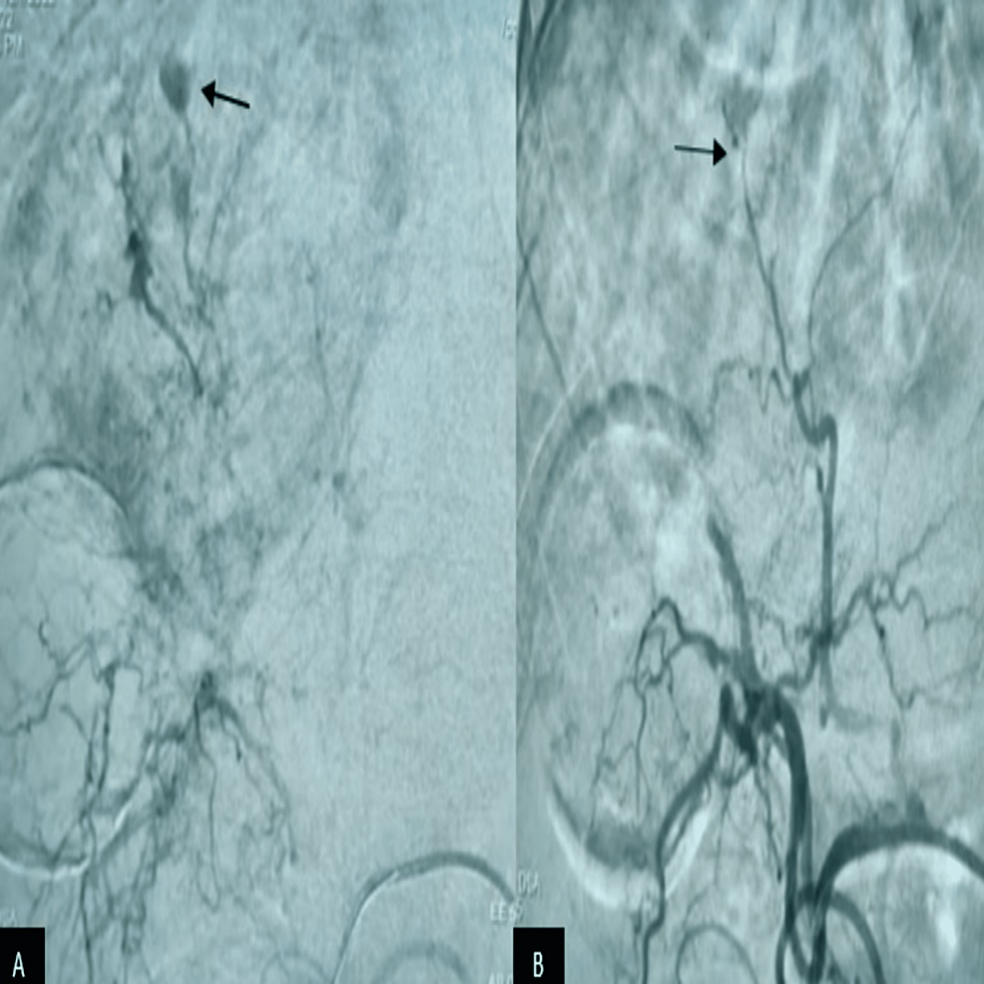
Celiac angiogram (A) before trans-arterial embolization (B) after trans-arterial embolization (see arrows)

The dilemma of managing NSTEMI in the presence of an intra-tumoral bleed led to performing a mesenteric celiac angiogram, that showed an abnormal hypervascularity with tumor blush from the hepatic artery coursing through the tumor in segment VIIII (Figure 4A). Of note, multiple medical conditions had to be considered while managing the patient. The diagnosis of NSTEMI with CCF required the management of pulmonary edema, along with administration of DAPTs and anticoagulation for a subsequent CAG. The presence of a co-existing tumoral bleed from the HCC could have aggravated the above line of management. Thus, the patient was first stabilized with dual diuretics and on was kept on Non-Invasive Ventilation, following which DAPTs and enoxaparin were started and an emergency (within 12 hours) invasive angiography was planned to trans-arterially embolize the bleeding tumoral vessel and perform the CAG in the same sitting. Trans arterial therapeutic embolization of the right hepatic artery was then performed via the femoral route, with polyvinyl alcohol particles (300-500micrometer). Celiac angiogram after embolization showed no tumor blush (Figure 4B).

**Figure 5:**
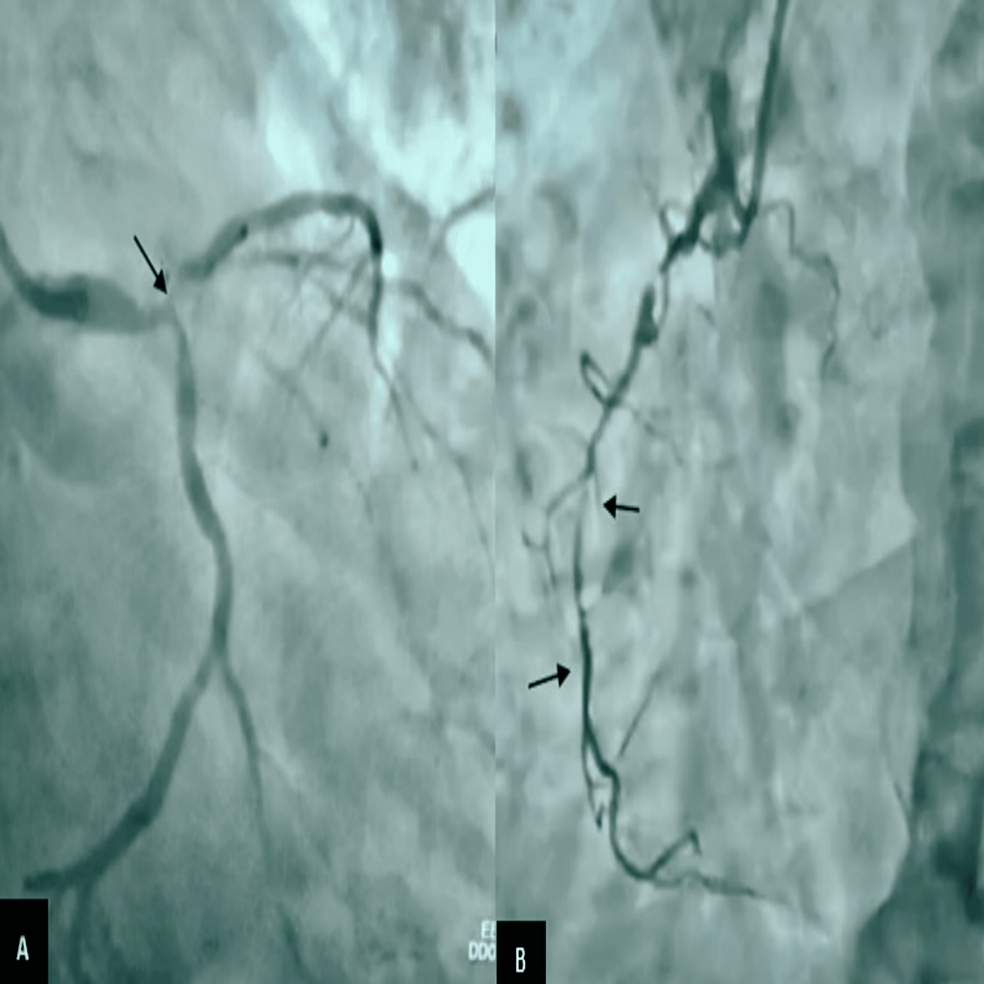
Pre-percutaneous transluminal coronary angioplasty (A) Left main artery critical distal stenosis with ostial LAD and LCX involvement; (B) diffusely diseased RCA (see arrows).

Additionally, the CAG performed through the femoral route revealed a triple vessel disease, with a diffusely diseased right coronary artery (RCA) in its whole course, 60% stenosis of the distal left main artery, 90% ostial involvement of the left anterior descending artery (LAD), and an 80% ostial involvement of the left circumflex artery (LCX) (Figure 5).

**Figure 6:**
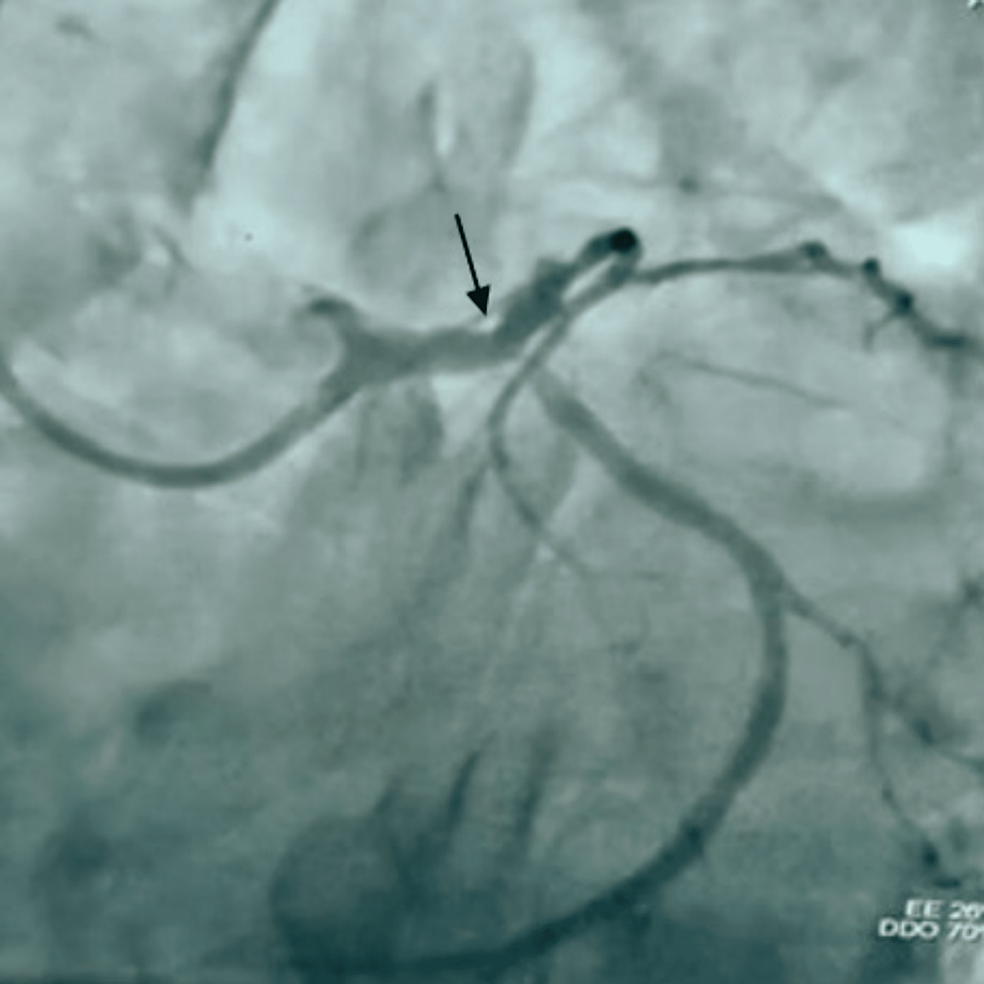
The left main artery over left anterior descending artery stenting post percutaneous transluminal coronary angioplasty

Therefore, in the same setting, a PTCA was performed with a 3.5x24mm drug-eluting stent (DES) in the left main artery through the right femoral access (Figure 6).

The patient was taken off bilevel positive airway pressure the following day and discharged after two days, following complete stabilization. He was further planned for endoscopic ultrasound-guided-fine needle aspiration (EUS-FNA) of the nodal metastasis with sorafenib therapy on follow-up [10-12].

## Discussion

This case is a suitable illustration of the multifaceted diagnostic workup and management of a 69-year-old male, with acute coronary syndrome and an incidental finding of hepatocellular carcinoma with active intra-tumoral bleeding. Incidental findings usually come with the challenge of deciding whether the further treatment for which the diagnostic workup has been performed, should be continued. In our case, multiple specialties were involved in deciding the further line of management. The patient’s acute presentation of an NSTEMI and CCF required immediate management of the patient and a diagnostic CAG was the next best step, for which DAPTs and anticoagulation had to be administered, but the coexisting tumoral bleed could have aggravated in that case. Hence, embolization of the hepatic artery along with administering diuretics, DAPTs, and anticoagulation seemed the next best approach.

Since there are no standard guidelines so far due to the rarity of such cases, the most up-to-date approach was hence followed. After thoroughly searching PubMed, PubMed Central, and Google Scholar, two cases with similar associations of acute coronary syndrome and hepatocellular carcinoma were found. One of the cases shows the occurrence of acute coronary syndrome occurring in a patient with hepatocellular carcinoma secondary to hepatitis C cirrhosis [13]. This differs from our current report as the patient tested negative for hepatitis virus serology. Another case found is of a 75-year-old-female reporting the occurrence of an acute coronary syndrome occurring in a patient treated with sorafenib for unresectable hepatocellular carcinoma [14]. However, this case reports the association after the administration of sorafenib unlike a simultaneous occurrence unrelated to sorafenib administration as discussed above. This was an atypical case with respect to multiple factors. There was an absence of any abdominal pain, melena, vomiting, weight loss, irregular bowel bladder habits, or any underlying known chronic liver disease even in its stage of nodal metastasis, making it difficult to diagnose. The liver showed a normal function and architecture, with no evidence of cirrhosis on imaging, which has been rare [15]. Another incidental finding observed, was that of a hydatid cyst. A coexistence of hydatid cyst and hepatocellular carcinoma has been rarely observed, and studies suggest the presence of a hydatid cyst may have a protective effect against the development of hepatocellular carcinoma, while more research is required to clearly confirm this finding [16]. The EUS-FNA further confirmed the nodal metastasis to abdominal lymph nodes, which is a common site of metastasis of hepatocellular carcinoma.

## Conclusions

This is a rare case of simultaneous management of an acute coronary syndrome by performing a left heart catheterization which requires anticoagulation, in the setting of an incidentally detected probable hepatocellular carcinoma with active intra-tumoral bleeding. This case also highlights the importance of considering the possibility of a malignancy, even in the absence of any symptoms and underlying risk factors. The simultaneous occurrence of an acute coronary syndrome with a probable hepatocellular carcinoma and a hydatid cyst encourages further research to study the possibility of an association between them. The above report is a good reminder for physicians to be vigilant of the abdominal cuts on chest scans. 

## References

[1] Llovet JM, Kelley RK, Villanueva A (2021). Hepatocellular carcinoma. Nat Rev Dis Primers.

[2] Kew MC (2014). Hepatocellular carcinoma: epidemiology and risk factors. J Hepatocell Carcinoma.

[3] de Rave S, Hussain SM (2002). A liver tumour as an incidental finding: differential diagnosis and treatment. Scand J Gastroenterol Suppl.

[4] Lai EC, Wu KM, Choi TK, Fan ST, Wong J (1989). Spontaneous ruptured hepatocellular carcinoma. An appraisal of surgical treatment. Ann Surg.

[5] Tan WC, Inoue K, AbdelWareth L (2020). The Asia-Pacific Society of Cardiology (APSC) expert committee consensus recommendations for assessment of suspected acute coronary syndrome using high-sensitivity cardiac troponin T in the emergency department. Circ J.

[6] Ronot M, Vilgrain V (2014). Hepatocellular carcinoma: diagnostic criteria by imaging techniques. Best Pract Res Clin Gastroenterol.

[7] (2022). National Cancer Institute. Regional lymph nodes. https://www.training.seer.cancer.gov/biliary/anatomy/lymph-nodes.html.

[8] UpToDate UpToDate (2022). Child-Pugh classification of severity of cirrhosis. UpToDate.

[9] (2022). MELD calculator. https://www.mayoclinic.org/medical-professionals/calculators/meld-model/itt-20434705.

[10] Forner A, Reig M, Bruix J (2018). Hepatocellular carcinoma. Lancet.

[11] Maida M, Orlando E, Cammà C, Cabibbo G (2014). Staging systems of hepatocellular carcinoma: a review of literature. World J Gastroenterol.

[12] Xia F, Wu LL, Lau WY (2016). Adjuvant sorafenib after heptectomy for Barcelona Clinic Liver Cancer-stage C hepatocellular carcinoma patients. World J Gastroenterol.

[13] Lema HM, Temgoua MN, Engonwei NM (2019). Acute coronary syndrome in patient with viral hepatitis: an underdiagnosed condition in Sub-Saharan Africa. Cardiology and Cardiovascular Research.

[14] Ndam AW, Helles ML, Kowo PM (2020). Acute coronary syndrome occurring in a Sub-Saharan patient treated with a reduced dose of sorafenib for an unresectable hepatocellular carcinoma: case report. Open J Gastroenterol.

[15] Naganuma H, Ishida H, Ogawa M, Sato T, Sageshima M, Suzuki K, Ohyama Y (2019). Hepatocellular carcinoma in otherwise sonographically normal liver. J Clin Ultrasound.

[16] Bo R, Yasen A, Shao Y (2020). Co-existence of hepatocellular carcinoma and cystic echinococcosis. Infect Agent Cancer.

